# HOW DRIVING STRESS, CONFIDENCE, AND THOUGHTS ABOUT DRIVING ALTERNATIVES AFFECT TRANSPORTATION PLANNING

**DOI:** 10.1093/geroni/igz038.940

**Published:** 2019-11-08

**Authors:** Jonathon Vivoda, Jiawei Cao, Annie Harmon

**Affiliations:** 1 Miami University, Oxford, Ohio, United States; 2 Washington University, St. Louis, Missouri, United States

## Abstract

Although many older adults will eventually not be able to safely drive themselves, comprehensive planning for a non-driving future is infrequent. To better understand the factors that predict transportation planning, the role played by several different aspects of driving were assessed. We analyzed whether stress about driving, enjoyment of driving, driving confidence, recent experience with negative driving events, and thoughts about how well other transportation modes could meet one’s future needs played a role in planning. Age, gender, race, and income were also controlled for in the models. More transportation planning was significantly associated with experiencing driving as more stressful and with reporting less confidence in one’s driving skills. Level of driving enjoyment and the number of concerning driving events experienced during the past year (e.g., crash, near crash, driving conversations, health issues, etc.) were not significantly related to planning. Respondents were also asked about how well other transport modes (e.g., rides with other drivers, buses, light rail, taxis, etc.) could meet their needs when they can no longer drive. This factor explained more of the variance in the planning outcome than any of the other factors, with a larger number associated with more planning. This result suggests that engaging in more planning may be associated with an openness to non-driving options, but it is unclear if that is a result of more planning, or a precursor to this activity.

